# Acute promyelocytic leukemia with FIP1L1::RARA fusion gene: The clinical utility of transcriptome sequencing and bioinformatic analyses

**DOI:** 10.3389/fonc.2022.1049473

**Published:** 2023-01-26

**Authors:** Guanghua Liu, Jiangwen Long, Yuyu Chen, Lingqian Li, Xisha Huan, Panpan Long

**Affiliations:** ^1^ Laboratory of Hematology, Hunan Provincial People’s Hospital, The First Affiliated Hospital of Hunan Normal University, Changsha, China; ^2^ Department of Blood Transfusion, Hunan Provincial People’s Hospital, The First Affiliated Hospital of Hunan Normal University, Changsha, China; ^3^ Hunan Cancer Hospital (the Affiliated Cancer Hospital of Xiangya School of Medicine, Central South University), Clinical Laboratory, Changsha, China; ^4^ Genetic Center, Changsha Jiangwan Maternity Hospital, Changsha, China

**Keywords:** acute promyelocytic leukemia, *FIP1L1::RARA*, transcriptome sequencing, bioinformatic analyses, differential expression genes

## Abstract

**Background:**

Acute promyelocytic leukemia (APL) is typically characterized by the presence of coagulopathy and the *PML::RARA* fusion gene. The *FIP1L1::RARA* has been reported as a novel fusion gene, but studies on its pathogenesis are limited.

**Objectives:**

A *FIP1L1::RARA* fusion in a child finally diagnosed as APL was reported. RNA sequencing (RNA-seq) of six patients (three cases of acute lymphoblastic leukemia (ALL), one case of myelodysplastic syndrome (MDS), one case of acute megakaryoblastic leukemia (M7), and one case of APL with *FIP1L1::RARA)* were performed.

**Methods:**

Transcriptome analysis of six patients was performed by RNA-seq. The heat map was used for showing the RNA expression profile, the volcano plot for identifying differential expression genes (DEGs), and the KEGG Orthology-Based Annotation System (KOBAS) online biological information database for KEGG pathway enrichment analysis.

**Results:**

Obvious differences between APL with *FIP1L1::RARA* and hematologic malignancies were identified. 1060 common differentially expressed genes (co-DEGs) were detected between APL with *FIP1L1::RARA* vs ALL and APL with *FIP1L1::RARA* vs myeloid neoplasms (MDS, M7), the up-regulated genes were mainly mapped into platelet activation, cancer, AMPK signaling pathway, PI3K-Akt signaling pathway, and MAPK signaling pathway. The down-regulated genes were significantly associated with TNF signaling pathway, Rap1 signaling pathway, Age-RAGE signaling pathway, and apoptosis.

**Conclusion:**

A *FIP1L1::RARA* fusion in a child finally diagnosed as APL was reported. RNA-seq may provide a new diagnostic method when *RARA* rearrangements fail to be identified by conventional methods. In the analysis of co-DEGs between case vs ALL and case vs myeloid neoplasms, the up-regulated and down-regulated genes were enriched in different signaling pathways. Further experimental studies are needed to identify pathogenesis and treatment for APL with *FIP1L1::RARA*.

## Introduction

Acute promyelocytic leukemia (APL) is characterized by abnormal promyelocytic proliferation and the presence of coagulopathy (1, 2). About 95% of APL is typically characterized by the fusion between promyelocytic leukemia (*PML*) and retinoic acid receptor alpha (*RARA*) and sensitive to differentiation induction therapy containing all-trans retinoic acid (ATRA) and arsenic trioxide (ATO) with a good clinical outcome. Rarely, about 2% of APL is characterized by atypical rearrangements with *PLZF, NPM1, NUMA, STAT5B, FIP1L1, PRKAR1A, BCOR, OBFC2A, TBLR1, GTF2I, IRF2BP2, FNDC3B, TFGA, NUP98, TNRC18, STAT3* and *TFG (* 3, 4). The *RARA* fusion partner plays an important role in the morphology and clinical features (5), alternative fusion types demonstrate varying sensitivity to ATRA (6).

*FIP1L1::RARA* is a rare alternative fusion type associated with myeloid neoplasm composed of 426 amino acids (AA) from the N terminus of *FIP1L1* and 403 AA from the C terminus of *RARA (* 7 *)*. 5 cases with *FIP1L1::RARA* have been reported in the literature at present. The *FIP1L1::RARA* fusion gene was first reported as a novel *RARA*-associated fusion gene in a 20-month-old child with juvenile myelomonocytic leukemia (JMML), no other mutations in *N-Ras, K-Ras*, or *PTPN11* were detected (8). In addition, a *FIP1L1::RARA* fusion gene was detected in a 90-year-old woman, who was finally diagnosed with APL and achieved complete remission after ATRA therapy alone (9). Another 77-year-old woman was also reported as APL with *FIP1L1::RARA* and the *FLT3-ITD*, *FLT3-D835Y*, *CEBPA* and *NPM1* mutations were not detected, *PML::RARA*, *AML1::ETO*, and *CBFβ::MYH11* fusion genes were negative. Unfortunately, she died after ten days of treatment according to Programa para el Tratamiento de Hemopatias Malignas (PETHEMA) APL 2005 protocol (10). Besides, a *FIP1L1::RARA* fusion was reported in a 28-month-old girl with APL who presented with an extramedullary tumor in the skull without the classic karyotype. An initial complete remission was achieved by a treatment combining ATRA with DA regimen (daunorubicin and cytarabine) and kept leukemia-free after a 5-month follow-up by continual therapy (11).

Despite reports of five patients with the *FIP1L1::RARA* fusion gene, among which three were diagnosed as APL, studies on its pathogenesis and treatment are limited. **Table 1** provides patient characteristics of all reported cases, including the current case. In this study, three cases of ALL and two cases of myeloid neoplasm were collected to explore the differences between APL with *FIP1L1::RARA* and other hematologic malignancies at the level of transcriptome and lay a certain molecular basis for further research on the treatment and prognosis.

**Table 1:** Characteristics of reported myeloid neoplasms with FIP1L1::RARA.

Citation	Age	Sex	FIP1L1	RARA	Karyotype	Mutation	Diagnosis	Sensitivity to ATRA
4	20-month-old	Male	exon 15	exon 3	46,XY,t(4;17)(q12;q21)[8]/46,XY[4]	no	JMML	no
5	90-year-old	Female	exon 15	exon 3	47,XX,t(4;17)(q12;q21),+8	unknown	APL	yes
6	77-year-old	Female	exon 13	exon 3	44,X,der(X)t(X);?(p)?;?,-2,-4,-16,+der(17)t(17);?(q21);?[cp10]	FLT3-ITD	APL	cannot assess
7	28-month-old	Female	exon 12	intron 3	46,XX,t(4;17)(q12;q22)[9]/46,idem,del(16)(q22)[3]/45,idem,-x,-4,-9,-15,del(16)(q22),+marl,+mar2,+mar3[7]/46,xx[3]	KRAS	APL	ATRA with DA
8	9-month-old	Male	exon 13	exon 3	unknown	MAP2K2p.R231L	MDS/MPN neoplasm overlap syndrome	ATRA with ATO
case	3-year-old	Female	exon 13	exon 2	47XX,t(4;17)(q12;21),+22(20)	no	APL	no

ARTA, all-trans retinoic acid; JMML, juvenile myelomonocytic leukemia; APL, acute promyelocytic leukemia; MDS, myelodysplastic syndrome; MPN, myeloproliterative neoplasms; ATO, arsenic trioxide; DA, daunorubicin+cytarabine.

## Materials and methods

### Patients

In total, six patients admitted to the pediatric department of Hunan Provincial People’s Hospital were included in this study, of which three were B-cell acute lymphoblastic leukemia (B-ALL, female, 4 years old).T he other three were myelodysplastic syndrome (MDS, male, 17 years old), acute megakaryoblastic leukemia (M7, female, 11 months old), and APL with *FIP1L1::RARA* (female, 4 years old). None of the six patients received neoadjuvant chemotherapy before transcriptome sequencing (RNA-Seq). This study was approved by Hunan Provincial People’s Hospital Medical Ethics Committee.

### RNA-Seq

PAXgene Blood RNA Tube (BD, cat.762165) was used for bone marrow sample collection. RNA was isolated by MagMAX for Stabilized Blood Tubes RNA Isolation Kit, compatible with PAXgene Blood RNA Tubes (Invitrogen, cat.4451894). NanodropOne (Thermofisher) was used for RNA concentration and purity. For RNA quality control, Qseq400 (BIOPTIC) with R1 cartridge (BIOPTIC), RQN≥5 is acceptable for following library preparation. Libraries were prepared with the KAPA RNA HyperPrep Kit (Kapabiosystems, cat. KK8540) after rRNA depletion with the KAPA RiboErase (HMR) Kit(Kapabiosystems, cat. KK8482), library preparation was started with 1ug total RNA per sample as described in kit handbook manually.

For Library quality control, Qubit 3.0 with qubit dsDNA HS Assay Kit (Thermofisher) was used for concentration, Qseq400 (BIOPTIC) with S2 cartridge (BIOPTIC) for library size, KAPA Library Quantification Kit (KAPA biosystems) for library quantification and novaseq6000, PE150, 15G raw data for sequencing.

### Flow cytometry, FISH studies, and cytogenetic analysis

Cytogenetic analysis of BM samples after short-term culture (24 h) was carried out according to a standard procedure. G-banded chromosome was determined according to International System for Human Cytogenetic Nomenclature. Commercially available *PML::RARa* dual color dual fusion DNA probes were used for FISH analysis.

### Molecular analysis

The primer sequences of the *FIP1L1::RARA* fusion gene are GTCCTTTCTGAAAGATCTGCTACTGAAGTAGACAACAATTTTAGCAAACCACCTCCGTTTTTCCCTCCAGGAGCTCCTCCCACTCACCTTCCACCTCCTCCATTTCTTCCACCTCCTCCGACTGTCAGCACTGCTCCACCTCTGATTCCACCACCGGCCATTGAGACCCAGAGCAGCAGTTCTGAAGAGATAGTGCCCAGCCCTCCCTCGCCACCCCCTCTACCCCGCATCTACAAGCCTTGCTTTGTCTGTCAGGACAAGTCCTCAGGCTACCACTATGGGGTCAGCGCCTGTGAGGGCTGCAAG. The primer sequences of the *WT1* gene are as follows: WT1 forword: ACAGGGTACGAGAGCGATAACCA; WT1 reverse: CACACGTCGCACATCCTGAAT; WT1 probe: CAACGCCCATCCTCTGCGGAGCCCA. The primer sequences of the *PML::RARA* fusion gene are as follows: bcr1-ENF903: TCTTCCTGCCCAACAGCAA; bcr2-ENF906: ACCTGGATGGACCGCCTAG; bcr3-ENF905: CCGATGGCTTCGACGAGTT; RARa-1099R ACATGCCCACTTCAAAGCAC. The primer sequences of the *CBFβ::MYH11 fusion* gene are as follows: CBFB-C: GGGCTGTCTGGAGTTTGATG; MYH11-D1:

TCCCTGTGACGCTCTCAACT; MYH11-D2: CTTGAGCGCCTGCATGTT. Illumina Hiseq X sequencing platform was used to detect RNA-level gene fusions and SNVs/Indels variants at the whole transcriptome level. The bioinformatics analysis methods of gene mutation and gene fusion are as follows: the sequencing fragment was compared with the EnsemblGRCh37 reference genome by STAR software. Mutation detection was performed by VarScan software, including mutation threshold screening and SNVs mutation detection and typing. The fusion gene was analyzed by Arriba software. Mutation results were annotated by AnnovAR for downstream data, and the annotation databases mainly included Clinvar, dbSNP, 1000 Genome, genomeAD, ExAC, COSMIC, etc. The fusion gene databases include but are not limited to COSMIC, Fusion can cer, Atlas of Genetics and Cytogenetics in Oncology and Haematology, My Cancer Genome, etc.

### Statistical analysis

Differential gene expression and heat maps were performed by the DESeq2 and pheatmap packages in R (1.16.1). Kyoto Encyclopedia of Genes and Genomes (KEGG) Orthology-Based Annotation System (KOBAS) (version 3.0) online biological information database was used for KEGG pathway enrichment analysis of differentially expressed genes (DEGs). At least log FC > 1.5 (up-regulated) or log FC < -1.5 (down-regulated) and adjusted P-value <0.05 were considered statistically significant.

**Figure 1:**
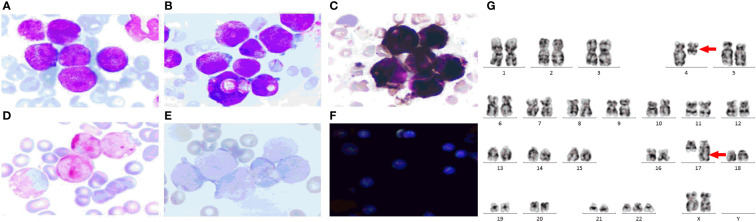
The clinical information of the APL patient. **(A)** Peripheral blood cell morphology at initial diagnosis. ×400; **(B)** Bone marrow morphology at initial diagnosis. ×400; **(C)** Peroxidase staining.×400; **(D)** Naphythol AS-D chloroacetate esterase staining.×400; **(E)** α-naphthol acetate esterace staining.×400; **(F)** FISH using the *PML::RARA* dual-color, dual fusion translocation probe. **(G)** The chromosome karyotype of the APL patient, the red arrows indicate the chromosome translocation. The *PML* probes are labeled with orange fluorescein and *RARA* probes with green fluorescein, the green and orange signal form a yellow fusion signal when *PML::RARA* fusion gene is present.

**Figure 2:**
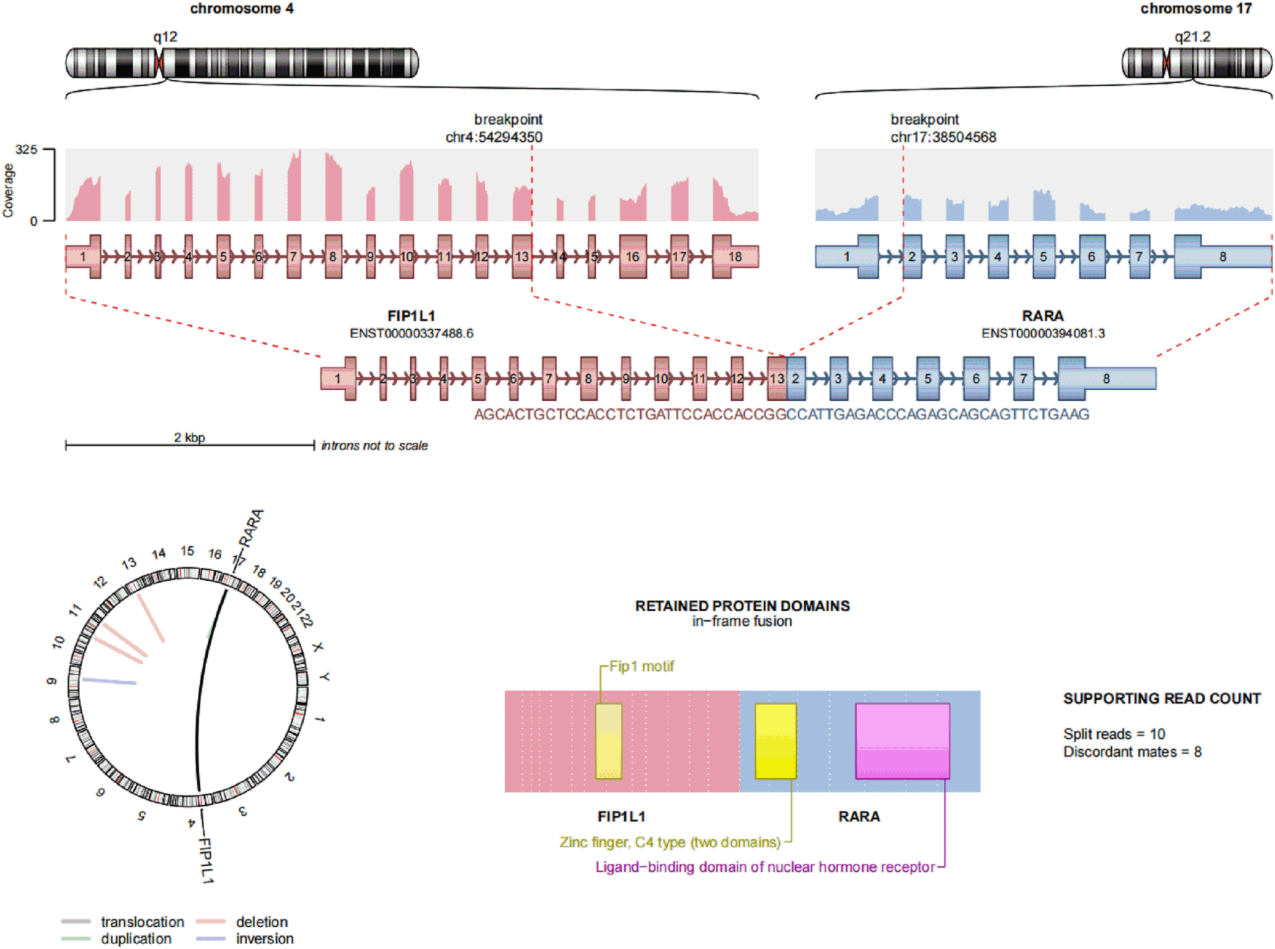
Transcriptome sequencing of *FIP1L1::RARA* fusion gene at onset, a fusion between exon 13 of *FIP1L1* and exon 3 of *RARA* was identified.

Case report: a 3-year-old girl presented with intermittent pain in her lower limb for half a month, and fever for 3 days. Physical examination was significant for scattered hemorrhagic spots on the face, mild hepatomegaly, and enlarged cervical lymph node. Brain magnetic resonance imaging showed inflammation or effusion in the bilateral middle ear mastoid process, and no obvious abnormalities in the brain. Initial laboratory evaluation revealed a high white blood cell (WBC) count of 246 × 10^9^/L, hemoglobin of 6.6g/dl, and a platelet count of 54 × 109/L. Coagulation function showed a prothrombin time of 15.6s, international normalized ratio of 1.38, activated partial thromboplastin time of 22.6s, thrombin time of 19.2s, fibrinogen at 2.61g/L, fibrinogen degradation products at 100.4 ug/ml, d-dimer at 37.76 mg/L, while lactate dehydrogenase was 4179 U/L. The peripheral blood cell differential count revealed a predominance of abnormal promyelocyte cells (60%) (**Figure 1A**). Bone marrow examination revealed 39.5% promyelocytes that had numerous hypergranularity without evidence of Auer rods (**Figure 1B**), the rate of positive peroxidase staining was 100% (**Figure 1C**), the rate of naphythol AS-D chloroacetate esterase (AS-D-CE) was 86% (**Figure 1D**), α-naphthol acetate esterace (α-NAE) was negative (**Figure 1E**). The immunophenotype was positive for MPO, CD33, CD13, CD15, CD123, CD38, and CD64 and negative for CD34, CD117, CD19, CD7, CD56, CD11b, and HLA-DR. Fluorescence *in situ* hybridization (FISH) analysis with the *PML::RARA* dual-fusion translocation probe identified no dual fusion signal (**Figure 1F**), and the *BCR::ABL*, *AML1::ETO*, and *CBFβ::MYH11*fusion genes were negative. Quantitative detection of the *WT1* gene showed it was highly expressed, and the positive rate was 98.2771%. Reverse transcriptase polymerase chain reaction (RT-PCR) revealed *PML::RARA* fusion gene (*Bcr1, Bcr2, Bcr3*) was negative. Besides, conventional karyotype analysis revealed a karyotype in 20 metaphase cells with the following formula: 47XX,t(4;17)(q12;21),+22(20) according to ISCN2016 (**Figure 1G**). To further characterize, RNA-seq was arranged for hematopoietic malignancies, sequencing of this sample confirmed the *FIP1L1::RARA* and *RARA::FIP1L1* fusion transcripts, the *FIP1L1::RARA* fusion gene was generated between exon 13 of *FIP1L1* and exon2 of *RARA* (**Figure 2**). Whole exon sequencing (WES) and copy number variation-sequencing (CNV-Seq) revealed dup(22)(q11.1q13.33), which can occur in hematologic malignancies such as acute myeloid leukemia (AML) and myelodysplastic syndrome (MDS). This patient was finally diagnosed with APL according to bone marrow morphology, immunophenotype, cytogenetics and RNA-seq. Besides, single-drug chemotherapies/chemotherapy regimens based on an ATP-based tumor chemosensitivity assay (ATP-TCA) were used to evaluate the *in vitro* chemosensitivity (**Table 2**). Tumor cells are moderately sensitive to single-drug chemotherapies including epirubicin, mitoxantrone, homoharringtonine, arsenic trioxide, selinexor, and chemotherapy regimens including FLAG (fludarabine and cytarabine), CLAG (clavibine and cytarabine) and ME (mitoxantrone and etoposide) and no sensitivity to ATRA. The body weight of the child was 16.3 kg, the height was 105 cm, and the body surface area was 0.69 m^2^. After a treatment combining ATRA (16.7 mg/d, divided twice a day) with hydroxycarbamide (1.5 g/d, divided three times a day) for 3 days, WBC count decreased from 246×10^9^ to 241×10^9^, indicating no response to ATRA. The protocol was changed to a FALG chemotherapy regimen (fludarabine 20 mg/d, Day1-Day5; cytarabine 1.3g/d, Day1-Day5; granulocyte colony-stimulating factor 80ug/d, Day0-Day7, idarbicin 5mg/d, Day4-Day6). After chemotherapy, the WBC of the patient dropped to 0.15 × 10^9^, suggested that the patient benefited from this chemotherapy. She received the same chemotherapy regimen after 40 days, and achieved a complete remission with minimal residue detection (MRD) negative. *FIP1L1::RARA* was negative by digital-PCR analysis. Conventional karyotype analysis revealed a normal karyotype in 20 metaphase cells. Quantitative detection of Wilms tumor 1 (*WT1)* gene showed the expression was high with a positive rate of 0.7548%. Then, she received an HAE chemotherapy regimen (homoharringatonine, 2 mg/d, Day1-Day5; cytarabine, 134 mg/d, Day1-Day5; etoposide, 67 mg/d, Day1-Day5) 30 days after the last chemotherapy, MRD detection showed no immunophenotypic abnormalities. *FIP1L1::RARA* was negative by digital-PCR analysis. Quantitative detection of *WT1* gene showed the expression was low, and the positive rate was 0.1636%. Last chemotherapy treatment, she received a MidAC chemotherapy regimen (mitoxantrone, 20 mg/d, Day1-Day5; cytarabine, 0.7g/d, Day1-Day3) 40 days after the last chemotherapy. MRD and *FIP1L1::RARA* fusion gene were negative. Quantitative detection of *WT1* gene showed the expression was high, and the positive rate was 0.7563%.

**Table 2:** Evaluating the *in vitro* chemosensitivity of single drug chemotherapies/chemotherapy regimens to APL with FIP1L1::RARA.

Drug therapy	Classification	Dose	Inhibition rate(%)	Sensitivity
Epirubicin	cytotoxic drugs	120 mg/m^2^	64.04	moderate
		60 mg/m^2^	57.69	moderate
Arsenic trioxide	cytotoxic drugs	7 mg/m^2^	62.13	moderate
Mitoxantrone	cytotoxic drugs	14 mg/m^2^	59.43	moderate
		8 mg/m^2^	52.75	moderate
Homoharringtonie	cytotoxic drugs	4 mg	53.07	moderate
		1 mg	25.68	low
Daunorubicin	cytotoxic drugs	90 mg/m^2^	28.31	low
		60 mg/m^2^	18.02	no
Tretinoin	cytotoxic drugs	10 mg	7.77	no
Selinexor	cytotoxic drugs	80 mg	54.73	moderate
		60 mg	49.32	low
Dasatinib	cytotoxic drugs	100 mg	21.96	low
FLAG	chemotherapy	Fludarabine 30 mg/m^2^ Cytarabine 2 g/m^2^	76.62	moderate
CLAG	chemotherapy	Cladribine 5 mg/m^2^ Cytarabine 2000 mg/m^2^	74.61	moderate
ME	chemotherapy	Mitoxantrone 10 mg/m^2^ Etoposide 100 mg/m^2^	71.14	moderate
DAT	chemotherapy	Daunorubicin 25 mg/m^2^ Cytarabine 100 mg/m^2^ mercaptopurine100 mg/m^2^	44.36	low

Highly sensitive: inhibition rate ≥ 80%; moderate sensitive: 50% ≤ inhibition rate < 80%; low sensitive: 20% ≤ inhibition rate < 50%; not sensitive: inhibition rate < 20%.

## Results

### Analysis of total RNA expression

**Figure 3:**
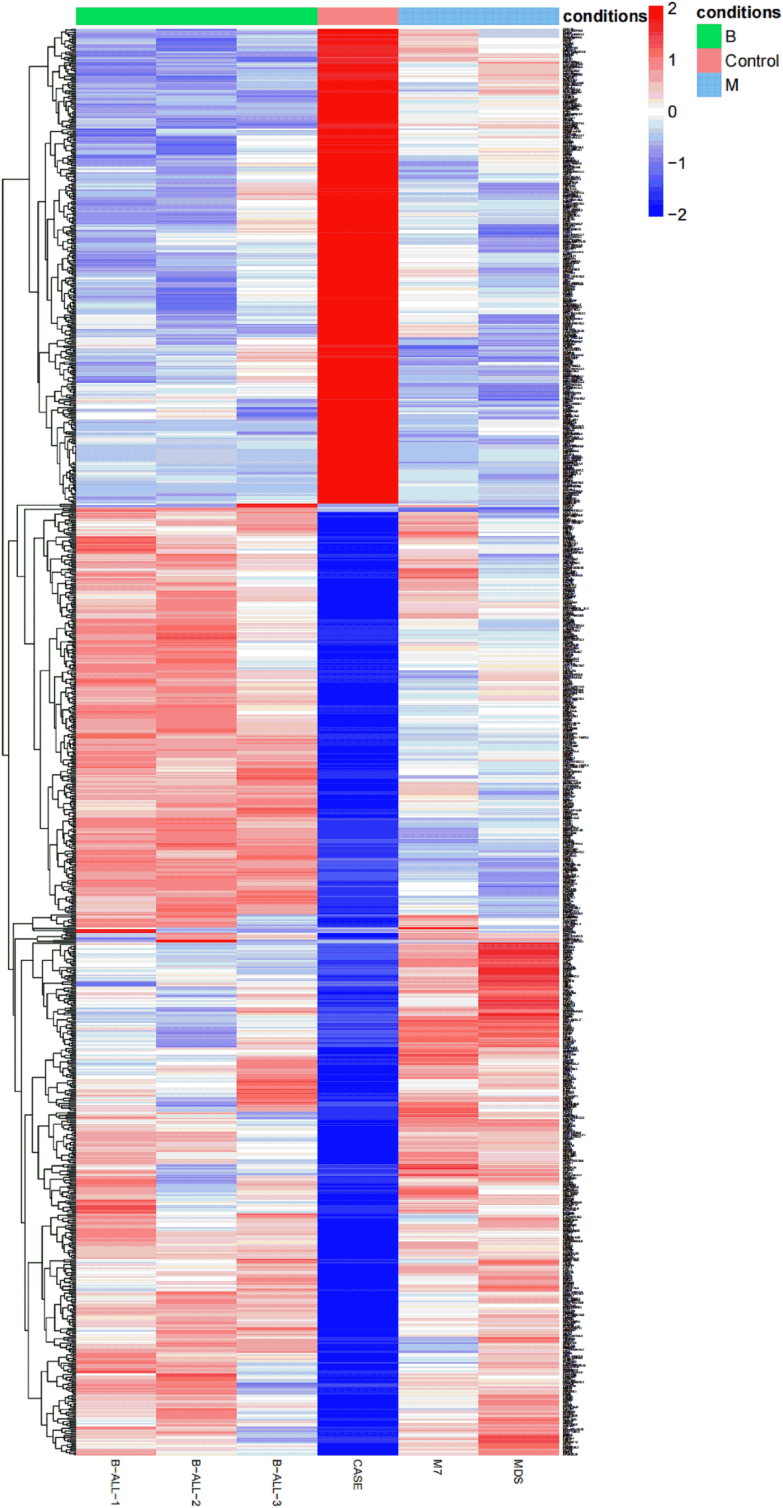
A heat map is created based on the total RNA expression of six patients with hematologic malignancies: the relative expression abundance of each gene in B-ALL, APL with *FIP1L1::RARA*, M7, MDS. The coloured scale varies from deep blue to deep red, which indicates low or high expression.

In order to understand the different RNA expression profile of each case, transcriptome analysis of six bone marrow specimens was performed by RNA-seq. The heat map was used to show the total RNA expression profile of each patient. Obvious differences between APL with *FIP1L1::RARA* and other hematologic malignancies were identified (**Figure 3**).

### Analysis of differentially expressed genes

**Figure 4:**
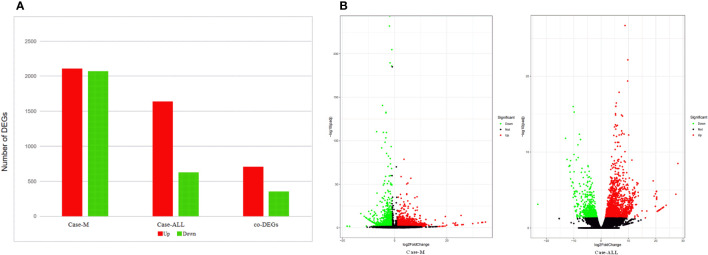
**(A)** DEGs between case group and M group, case group vs ALL group, co-DEGs between case vs ALL and case vs M; **(B)** Volcano map of DEGs. The x-axis is the log2 scale of the fold change of gene expression in case, ALL and M (log2(fold change)). Negative values indicate down regulation; positive values indicate up regulation. The y-axis is the minus log10 scale of the adjusted p values (elog10 (padj)), which indicates the significant level of expression difference. The red dots represent significantly up-regulated genes with at least two fold change, while the green dots represent significantly down-regulated genes with at least two fold change.

To identify the DEGs between different hematological malignancies, the patients were separated into three groups: case group (APL with *FIP1L1::RARA*), ALL group (three cases of B-ALL), and myeloid neoplasms (M) group (M7 and MDS). After analyzing the transcriptomic changes of the case group and M group, a total of 4181 DEGs (|fold change>2| and p<0.05) were identified, including 2110 up-regulated and 2071 down-regulated genes. Between the case group vs ALL group, 2268 DEGs were observed, among which 1639 were up-regulated genes and 629 were down-regulated genes. A total of 1060 common DEGs (co-DEGs) were identified between case vs ALL and case vs M, including 706 up-regulated and 354 down-regulated genes (**Figure 4A**). To make it more intuitive, the volcano plot was used for identifying DEGs (**Figure 4B**).

### Enrichment and visualization of signaling pathways

**Figure 5:**
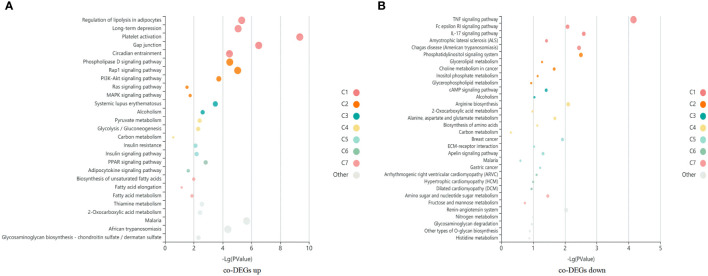
A KEGG pathway enrichment analysis was conducted to explore the most significantly enriched pathways for co-DEGs. Each bubble represents an enriched function, and the size of the bubble from small to large: [0.05,1], [0.01,0.05), [0.001,0.01), [0.0001,0.001), [1e-10,0.0001), [0,1e-10). The color of the bar is the same as the color in the circular network, which represents different clusters. For each cluster, if there are more than 5 terms, top 5 with the highest enrich ratio will be displayed. **(A)** KEGG enrichment bubble map of co-upregulated differential genes in case compared with M and ALL groups. **(B)** KEGG enrichment bubble map of co-downregulated differential genes in case compared with M and ALL groups.

A total of 1060 co-DEGs (|fold change|>2 and p < 0.05) were detected between case vs ALL and case vs M, a KEGG pathway enrichment analysis was conducted to explore the most significantly enriched pathways for co-DEGs. The up-regulated genes were mainly mapped into platelet activation, cancer, AMPK signaling pathway, PI3K-Akt signaling pathway, and MAPK signaling pathway. The down-regulated genes were significantly associated with TNF signaling pathway, Rap1 signaling pathway, Age-RAGE signaling pathway, and apoptosis (**Figure 5**).

## Discussion

In this study, a patient with the *FIP1L1::RARA* fusion gene was reported and eventually diagnosed as APL. Only the *FIP1L1::RARA* fusion gene was identified, with no evidence of an established oncogenic RAS pathway mutation. Although APL with atypical rearrangements usually has a clinical presentation and morphological and immunophenotypic picture similar to classical APL, several different features exist compared with classical APL including very young age, and absence of laboratory of coagulopathy.

Due to there being few reports on the *FIP1L1::RARA* fusion gene, studies on the pathogenesis, treatment, and prognosis of APL with *FIP1L1::RARA* are limited. Since it is an aggressive disease (12), there exists a high possibility of early mortality if clinicians are unable to identify an abnormal karyotype that appeared singularly or as part of a complex mutation (13). Identifying variant *RARA* rearrangements is critical for the diagnosis and treatment of patients with APL (14). In order to lay a molecular basis for further research on the occurrence and treatment of this disease, RNA-Seq was conducted to explore the differences between APL with *FIP1L1::RARA* and other hematologic malignancies from the level of transcriptome. Obvious transcriptomic differences between APL with *FIP1L1::RARA* and myeloid neoplasms were identified. In an analysis of co-DEGs, the up-regulated and down-regulated genes were enriched in different signaling pathways.

Many genes involved in APL have similar cellular functions, but lack recurrence and consistency, except for *FLT3*, *WT1*, and *KRAS.* The physical and functional interactions of the mutated genes have been useful to discover driver mutations in cancers, the damage of these genes in APL is weaker than the interaction between mutant genes and different functionally related classes (15). In this case, the molecular basis behind APL has been focused on the role of the *FIP1L1::RARA* fusion gene. The study indicated the retinoic acid receptor alpha (RARα) forms a heterodimer with another form of a nuclear hormone receptor protein called retinoid X receptors (RXR), the RARα- RXR heterodimer binds to regions of DNA referred to as retinoic acid response elements (RAREs) to mediate the transcription of hundreds of genes. Many of the RAREs are involved in self-renewal and differentiation (16). *RARA* has been implicated as a key regulator of normal as well as transformed blood cells, and APL is driven by fusion genes involving retinoic acid receptors, primarily *RARA*. Besides, *RARA* breakpoints are highly fixed in variant APL, mainly responsible for regulating bone marrow microenvironment and hematopoietic function. The constant involvement of *RARA* or other retinoic acid receptor super family members stresses the importance of deregulated retinoic acid signaling in driving APL pathogenesis (17). *FIP1L1* is an integral subunit for cleavage and polyadenylation specificity factor for stimulation of poly(A) polymerase. It was found that the *FIP1L1* is necessary for activating STAT5 and PKB/Akt in human hematopoietic progenitor cells (18), indicating it is a contributing factor to the higher proliferating activity. As for the roles and mechanisms of these genes in the different signaling pathways, further studies are needed to elucidate.

In therapy, patients with *FIP1L1::RARA* present different drug sensitivities to ATRA. A 90-year-old woman was reported as APL with *FIP1L1::RARA* fusion achieving a complete remission by oral administration of ATRA alone in a dose of 50 mg daily without further follow-up information, indicating a high sensitivity to ATRA (9). Another 77-year-old woman reported as APL with *FP1L1::RARA* fusion gene was treated according to the PETHEMA APL 2005 protocol. Unfortunately, she died after ten days of treatment, probably due to retinoic acid syndrome, and response to ATRA treatment could not be assessed in this case due to early death (10). A 28-month-old girl with *FIP1L1::RARA* and complex karyotype was diagnosed with APL complicated by the *de novo* myeloid sarcoma. After a treatment combining ATRA with DA regimen as induction chemotherapy, the patient had achieved an initial complete remission by day 30. However, *FIP1L1::RARA* was still positive after the second course. Then she received three cycles of ATRA and idarubicin in the following consolidation treatment and continual therapy with ATRA with chemotherapy as previously described and kept leukemia-free after a 5-month follow-up (11). During this case, the efficacy of the combination ATRA-chemotherapy therapy is positive, but the response to ATRA could not be assessed. These three patients with *FIP1L1::RARA* fusion were eventually diagnosed as APL. Several patients with *FIP1L1::RARA* were diagnosed as other myeloid neoplasms. A 20-month-old child with the *FIP1L1::RARA* fusion gene was first reported with a final diagnosis of JMML (8). Despite two allogeneic stem cell transplantations, the patient still died from relapse of JMML 17 months after presentation. No ATRA sensitivity was tested and there was no material to perform *in vitro* ATRA-mediated differentiation assays in this case. A 9-month-old boy with a *FIP1L1::RARA* fusion-associated myelodysplastic/myeloproliferative neoplasm-like overlap syndrome was also reported. After combined chemotherapy (ATRA, ATO, and idarubicin) and differentiating agents (ATRA, ATO), he achieved cytogenetic remission. After a consolidation cycle (mitoxantrone and cytarabine) and maintenance therapy (ATRA, ATO), he replased with new-onset facial swelling and palpable skull-based masses before allogeneic hematopoietic stem cell transplant (allo-HCT). Then he received achieved a cytogenetic remission after the Capizzi II regimen (high-dose cytarabine) reinduction therapy and topotecan, vinorelbine, thiotepa, and clofarabine chemotherapy combined with the synthetic retinoid agent tamibarotthis. After that, he proceeded to a peripheral blood stem cell transplant and achieved remission 20 months after allo-HCT (19). In this study, a 3-year-old girl with the *FIP1L1::RARA* fusion gene was eventually diagnosed as APL. At the beginning, she received ATRA treatment with no reducing of WBC, indicating no sensitivity to ATRA. Then she underwent a chemotherapy regimen for AML (two courses of FALG, one course of HAE, one course of MidAC). She achieved cytogenetic remission during every course, indicating high sensitivity to this chemotherapy regimen.

Reasons for patients with the *FIP1L1::RARA* fusion gene showing different sensitivities to ATRA are poorly understood. Study shows *FIP1L1::RARA* functions as a transcriptional repressor in retinoic acid-dependent transcription and can form a homodimer and suppress retinoic acid-dependent transcriptional activity, resulting in low sensitivity to ATRA (9). *FIP1L1::RARA* functions as a transcriptional repressor in retinoic acid-dependent transcription, FIP1 motif is a protein interaction domain and plays a pivotal role in homodimerization, which is crucial for retinoic acid-dependent transcriptional activity (20). Further studies are needed to elucidate the reasons for patients with the *FIP1L1::RARA* fusion gene exhibiting different drug sensitivities to ATRA. Since APL with *FIP1L1::RARA* has demonstrated different sensitivities to ATRA, combined chemotherapy rather than differentiation induction therapy may be the appropriate treatment for APL with *FIP1L1::RARA* with no sensitivity to ATRA. Reasons for different phenotypes of leukemia caused by *FIP1L1::RARA* remain unknown. Study indicates *FIP1L1::RARA* may interfere with *FIP1L1* function, resulting in leukemia with monocytic features. *FIP1L1* promoter regulated expression may influence *FIP1L1::RARA*-mediated leukemogenesis. Low-level expression of the fusion protein may have contributed to the non-APL phenotype (8). Moreover, the homodimerization ability of *RARA* fusion proteins is also critical for leukemic transformation (21). The different signal pathways enriched in this case may be the mechanism of *FIP1L1-RARA* leading to different types of leukemia in different ways, further studies are needed to elucidate the mechanism of different phenotypes of leukemia caused by *FIP1L1::RARA.* Since not all patients with *FIP1L1::RARA* are APL, it is important to combine morphology with flow cytometry, karyotype analysis, FISH, and RT-PCR for accurate diagnosis. In practice, when the APL phenotype was presented with a negative *PML::RARA* fusion gene and the translocation cannot be identified by conventional methods, APL with variant rearrangements of *RARA* needed to be considered and diagnosed as early as possible by fusion gene screening and RNA-Seq. RNA-seq may be a new diagnostic method when *RARA* rearrangements are failed to be identified by conventional methods, because of the obvious transcriptomic difference between APL with *FIP1L1::RARA* and myeloid neoplasms.

In this study, there are several limitations that need to be clarified. Firstly, there is no clinical data regarding ATO sensitivity, so whether the patient with *FIP1L1::RARA* will respond to ATO remains unknown. Secondly, the number of APL patients with *FIP1L1::RARA* and other myeloid neoplasms is extremely limited, and the credibility of the results is limited. In the follow-up work, the collection of cases and various subtypes will be expanded, further experimental verification will be carried out, and a diagnostic model based on transcriptome is expected to be constructed. Thirdly, the *in vitro* study data to confirm the association of *FIP1L1::RARA* with APL was insufficient. We plan to construct the expression plasmid and transfect to establish a stable cell line, further studying the association of *FIP1L1::RARA* with APL and the mechanism of APL induced by the *FIP1L1::RARA* fusion gene. Besides, we will explore the differences in different fusion sites and construct the cell models of a single agent/therapeutic regimen intervention *in vitro* studying the mechanism of drug resistance of APL to ATRA.

In conclusion, a child with *FIP1L1::RARA* fusion diagnosed as APL was reported. RNA-seq may be a new diagnostic method for patients with other *RARA* rearrangements. The up-regulated and down-regulated co-DEGs between case vs ALL and case vs myeloid neoplasms were enriched in different signaling pathways. Further experimental studies are needed to identify pathogenesis and treatment for APL with *FIP1L1::RARA*

## Data availability statement

The datasets presented in this study can be found in online repositories. The names of the repository/repositories and accession number(s) can be found below: BioProject, accession number PRJNA911738.

## Ethics statement

The studies involving human participants were reviewed and approved by Hunan Provincial People's Hospital Medical Ethics Committee. Written informed consent to participate in this study was provided by the participants’ legal guardian/next of kin. Written informed consent was obtained from the minor(s)’ legal guardian/next of kin for the publication of any potentially identifiable images or data included in this article.

## Author contributions

GL and PL developed the study design; GL wrote the original draft. PL reviewed the manuscript; YC was responsible for data acquisition and revising; JL, LL, and XH were responsible for data analysis and methodology. All authors approved the final version to be published.

## References

[1] Hambley BC Tomuleasa C Ghiaur G. Coagulopathy in acute promyelocytic leukemia: Can we go beyond supportive care? Front Med (2021) 8:722614. doi: 10.3389/fmed.2021.722614 PMC8415964 34485349

[2] Xiao MY Zhou P Liu YH Wei SJ Li D Li WY. Predictive factors for differentiating thrombohemorrhagic disorders in high-risk acute promyelocytic leukemia. Thromb Res (2022) 210:33–41. doi: 10.1016/j.thromres.2021.12.020 34998209

[3] Zhang X Sun JW Yu WJ Jin J. Current views on the genetic landscape and management of variant acute promyelocytic leukemia. biomark Res (2021) 9(1):33. doi: 10.1186/s40364-021-00284-x 33957999 PMC8101136

[4] Guarnera L Ottone T Fabiani E Divona M Savi A Travaglini S. Atypical rearrangements in APL-like acute myeloid leukemias: Molecular characterization and prognosis. Front Oncol (2022) 12:871590. doi: 10.3389/fonc.2022.871590 35494081 PMC9039303

[5] Nakanishi T Nakaya A Nishio Y Fujita S Satake A Azuma Y. A variant of acute promyelocytic leukemia with t(4;17)(q12;q21) showed two different clinical symptoms. Hematol Rep (2019) 11(3):7971. doi: 10.4081/hr.2019.7971 31579135 PMC6761461

[6] Baba SM Pandith AA Shah ZA Baba RA. Pathogenetic implication of fusion genes in acute promyelocytic leukemia and their diagnostic utility. Clin Genet (2019) 95(1):41–52. doi: 10.1111/cge.13372 29700805

[7] Leguit RJ Orazi A Kucine N Kvasnicka HM Gianelli U Arber DA. EAHP 2020 workshop proceedings, pediatric myeloid neoplasms. Virchows Archiv (2022) 481(4):621–46. doi: 10.1007/s00428-022-03375-8 PMC9534825 35819517

[8] Buijs A Bruin M. Fusion of FIP1L1 and RARA as a result of a novel t(4;17)(q12;q21) in a case of juvenile myelomonocytic leukemia. Leukemia (2007) 21(5):1104–8. doi: 10.1038/sj.leu.2404596 17301809

[9] Kondo T Mori A Darmanin S Hashino S Tanaka J Asaka M. The seventh pathogenic fusion gene FIP1L1::RARA was isolated from a t(4;17)-positive acute promyelocytic leukemia. Haematologica (2008) 93(9):1414–6. doi: 10.3324/haematol.12854 18603554

[10] Menezes J Acquadro F Perez-Pons de la Villa C García-Sánchez F Álvarez S Cigudosa JC. FIP1L1::RARA with breakpoint at FIP1L1 intron 13: a variant translocation in acute promyelocytic leukemia. Haematologica (2011) 96(10):1565–6. doi: 10.3324/haematol.2011.047134 PMC3186322 21750086

[11] Wang YR Rui YY Shen Y Li J Liu PN Lu Q. Myeloid sarcoma type of acute promyelocytic leukemia with a cryptic insertion of RARA into FIP1L1: The clinical utility of NGS and bioinformatic analyses. Front Oncol (2021) 11:688203. doi: 10.3389/fonc.2021.688203 34249738 PMC8264125

[12] Jiang XW Chen SZ Zhu XY Xu XX Liu Y. Development and validation of a droplet digital PCR assay for the evaluation of PML-RARα fusion transcripts in acute promyelocytic leukemia. Mol Cell Probes (2020) 53:101617. doi: 10.1016/j.mcp.2020.101617 32585184

[13] Chen C Huang X Wang K Chen K Gao D Qian S. Early mortality in acute promyelocytic leukemia: Potential predictors. Oncol Lett (2018) 15(4):4061–9. doi: 10.3892/ol.2018.7854 PMC5835847 29541170

[14] Peterson JF He RR Nayer H Cuevo RS Smadbeck JB Vasmatzis G. Characterization of a rarely reported STAT5B/RARA gene fusion in a young adult with newly diagnosed acute promyelocytic leukemia with resistance to ATRA therapy. Cancer Genet (2019) 237:51–4. doi: 10.1016/j.cancergen.2019.06.007 31447065

[15] Ibáñez M Carbonell-Caballero J García-Alonso L Such E Jiménez-Almazán J Vidal E. The mutational landscape of acute promyelocytic leukemia reveals an interacting network of Co-occurrences and recurrent mutations. PLoS One (2016) 11(2):e0148346. doi: 10.1371/journal.pone.0148346 26886259 PMC4757557

[16] Jimenez JJ Chale RS Abad AC Schally AV. Acute promyelocytic leukemia (APL): a review of the literature. Oncotarget (2020) 11(11):992–1003. doi: 10.18632/oncotarget 32215187 PMC7082115

[17] Geoffroy MC de Thé H. Classic and variants APLs, as viewed from a therapy response. Cancers (Basel) (2020) 12(4):967. doi: 10.3390/cancers12040967 32295268 PMC7226009

[18] Buitenhuis M Verhagen LP Cools J Coffer PJ. Molecular mechanisms underlying FIP1L1-PDGFRA-mediated myeloproliferation. Cancer Res (2007) 67(8):3759–66. doi: 10.1158/0008-5472 17440089

[19] Miltiadous O Petrova-Drus K Kaicker S Mathew S Kluk M Geyer JT. Successful treatment and integrated genomic analysis of an infant with FIP1L1::RARA fusion-associated myeloid neoplasm. Blood Adv (2022) 6(4):1137–42. doi: 10.1182/bloodadvances.2021004966 PMC8864666 34551074

[20] Iwasaki J Kondo T Darmanin S Ibata M Onozawa M Hashimoto D. FIP1L1 presence in FIP1L1::RARA or FIP1L1-PDGFRA differentially contributes to the pathogenesis of distinct types of leukemia. Ann Hematol (2014) 93(9):1473–81. doi: 10.1007/s00277-014-2085-1 24763514

[21] Licht JD. Reconstructing a disease: What essential features of the retinoic acid receptor fusion oncoproteins generate acute promyelocytic leukemia? Cancer Cell (2006) 9(2):73–4. doi: 10.1016/j.ccr.2006.01.024 16473273

